# Development of the Low-Pressure Die Casting Process for an Aluminium Alloy Part

**DOI:** 10.3390/ma17122835

**Published:** 2024-06-10

**Authors:** Filipe Monteiro, Gonçalo Soares, Rui Madureira, Rui Pedro Silva, José Silva, Rui Amaral, Rui Neto, Ana Reis, António Esteves

**Affiliations:** 1 INEGI—Institute of Science and Innovation in Mechanical and Industrial Engineering, Rua Dr. Roberto Frias, 4200-465 Porto, Portugal; gsoares@inegi.up.pt (G.S.); rdmadureira@inegi.up.pt (R.M.); rpsilva@inegi.up.pt (R.P.S.); jmsilva@inegi.up.pt (J.S.); ramaral@inegi.up.pt (R.A.); rneto@inegi.up.pt (R.N.); arlr@fe.up.pt (A.R.); 2 Department of Mechanical Engineering, Faculty of Engineering, University of Porto, Rua Dr. Roberto Frias, 4200-465 Porto, Portugal; 3 FAB–Fundição de Alumínios de Braga, Parque Industrial Sobreposta, Lugar da Alagoa, Este (São Pedro e São Mamede), 4715-533 Braga, Portugal; antonio.esteves@fab.pt

**Keywords:** low-pressure die casting, AlSi7Mg0.3, ProCAST^®^, lightweight, mobility

## Abstract

The low-pressure die casting (LPDC) process was experimentally and numerically studied to produce AlSi7Mg0.3 components such as steering knuckles. Steering knuckles are important safety components in the context of a vehicle’s suspension system, serving as the mechanical interface that facilitates the articulation of the steering to control the front wheel’s orientation, while simultaneously bearing the vertical load imposed by the vehicle’s weight. This work focuses on the development of a numerical model in ProCAST^®^, replicating the production of the aforementioned part. The model analyses parameters such as the filling dynamics, solidification process, and presence of shrinkage porosities. For the purpose of evaluating the quality of the castings, six parts were produced and characterised, both mechanically (tensile and hardness tests) and microstructurally (porosity and optical microscopy analysis). When correlating simulation results with the available experimental data, it is possible to conclude that the usage of the LPDC process is a viable alternative to the use of steels and other metals for the production of very high-quality castings while using lighter alloys such as aluminium and magnesium in more demanding applications.

## 1. Introduction

The steering knuckle is a critical component within a vehicle’s suspension system, serving multiple essential functions. Within the automotive industry, steering knuckles require attributes such as exceptional mechanical strength, unyielding structural rigidity, and a propensity for lightweight construction [1,2,3]. This need arises from the mechanics of steering, whereupon the actuation of the steering wheel by drivers induces tensile forces upon one portion of the steering knuckle and compressive forces upon the other. These forces are generated due to the rotational dynamics of the wheel, resulting in torsional loads upon the steering knuckle [4,5].

In the automotive manufacturing context, the fabrication of steering knuckles is performed through two primary methodologies: forging and casting. Notably, castings are prone to the presence of blowholes, which are deleterious in terms of fatigue resistance and overall durability, with forging providing overall more structurally robust components [6,7].

For the material selection, due to the previously mentioned mechanical requirements, the typically used materials in the production of steering knuckles are low/medium carbon steels, low-alloy steels, ductile cast iron, and aluminium alloys, with the optimal material selection depending on the specific conditions the steering knuckle is expected to endure [6]. Nowadays most industries are pushing to increase the usage of aluminium alloys as the material of choice, due to their characteristic low weight and consequent ability to offer a possible reduction in fuel consumption and CO_2_ emissions [6,7,8].

The challenges and requirements described in the last few paragraphs are not just limited to the automotive industry but are also felt by other demanding sectors, such as the motorbike industry, for structural parts.

When considering aluminium alloys for applications such as structural and safety components, the methods of choice fall to low-pressure die casting (LPDC) and derivative processes such as counter-pressure casting (CPC) [9,10] due to their known capabilities of producing high-performance castings where the presence of defects such as porosities is very reduced [11]. A356, also known as AlSi7Mg0.3, in its heat-treated form (A356-T6), is the most frequently used low-pressure die-casting aluminium alloy for automotive loadbearing components, such as steering knuckles, due to its favourable properties, including good casting, machining, weldability, and positive mechanical properties [12,13].

**Figure 1:**
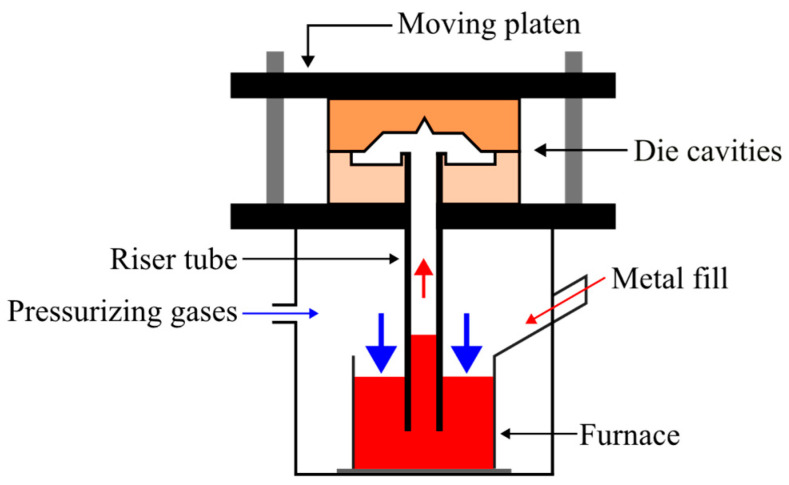
Schematic representation of an LPDC system, adapted from [15].

LPDC is a technique with growing adoption in the foundry industry for producing high-performance light alloy castings [11] with its primary application being the production of automotive wheels. LPDC is a counter-gravity pouring system that involves the upward displacement of molten metal into a cavity by applying controlled pressure to the metal surface within a hermetically sealed reservoir [14], as depicted in Figure 1.

The process offers several advantages, including a precisely controlled [16], non-turbulent fill that eliminates air entrapment almost entirely [17] and excellent feeding capacity [18], with the ready supply of molten metal in the direction of solidification significantly reducing the risk of shrinkage porosity associated with solidification [11,17,19,20]. These conditions greatly increase the mechanical properties of the casted parts and reduce the price of components, due to the minimisation of risers.

The versatility of this process is evident in its wide range of applications, spanning industries such as automotive manufacturing and machine tool production [21]. The term “low pressure” refers to the restricted pressure parameters applied in the process, typically ranging from 0.3 to 1.5 bar [22]. For example, in aluminium alloy casting, pressure levels around 0.58 to 0.60 bar are commonly used [14]. In some cases, higher pressures, around 1 bar, are employed to reduce porosity formation during casting [16].

**Figure 2:**
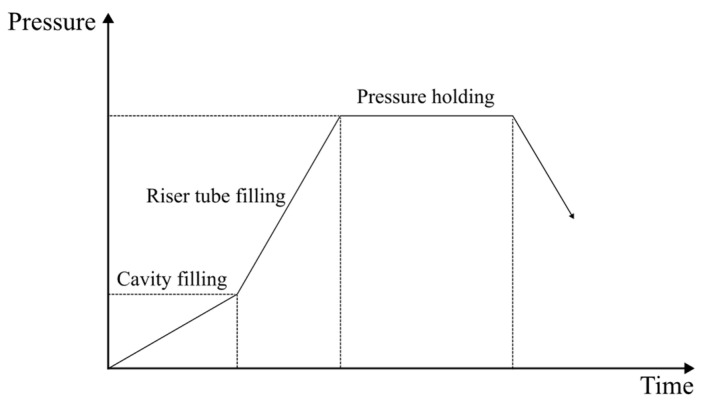
Typical pressure curve of a LPDC process, adapted from [15].

This process can be divided into three distinct phases [11,15], the riser tube filling, the cavity filling, and the pressure holding phases, as illustrated in Figure 2.

The usage of numerical simulation software like ProCAST^®^ for the modelling of casting processes has been essential in the development of the casting industry, diminishing drastically the duration required for the design and manufacturing of end products, enhancing the product quality through the examination of multiple manufacturing technology variations in a virtual environment, and results in cost savings related to technology development. ProCAST^®^ can comprehensively track the entire casting process, including mould filling, alloy cooling and crystallisation, defect formation, residual stress growth, and casting distortion [23,24,25,26].

The focus of this work is the production of steering knuckles from an AlSi7Mg0.3 aluminium alloy, using the low-pressure die casting process and the numerical simulation software ProCAST^®^ 18.0 (ESI Group, Madrid, Spain) incorporated with microstructural and mechanical characterisation of the produced castings to assess their quality and mechanical properties, with the objective to research on the applicability and repeatability of the process for the production of loadbearing components using light alloys.

## 2. Materials and Methods

### 2.1. Materials

The aluminium alloy used in this work was the AlSi7Mg0.3. Its chemical composition is presented in Table 1, alongside a comparison with the standard composition expected for a commercial-grade AlSi7Mg0.3 alloy. This alloy has a relatively low melting point (≈ 610 °C) with a temperature of 720 °C being the used casting temperature to ensure that no material falls under the liquidus temperature during the riser tube filling and cavity filling parts of the process. The temperature of the liquid metal was stabilised at around 740 °C during the melt preparation using a resistance furnace. The Mg contents were adjusted into the range of 0.45–0.5 wt.% with the addition of 2500 ppm pure Mg ingots, in anticipation of the Mg burnout phenomenon [27]. In the following step, the Sr content of the melt was increased up to 0.025 wt.% by adding 250 ppm AlSr10 master alloy rods in order to promote eutectic silicon modification [28,29]. Prior to casting, the melt was degassed with recourse to an FDU-2091 Mini-Degasser rotor degassing system (Foseco, Tamworth, England). The rotor was operated for 10 to 15 min at a speed of 80 rpm during the descending phase and 350 rpm during the degassing stage. The gas used for the degassing process was nitrogen, at a flow rate of 8 L/min during the descending phase and 15 L/min during the degassing stage. Posteriorly, the melt was cleaned to remove the slag formed, concluding the melt preparation process. A sample was extracted from the melt to determine its final composition with the Ametek SPECTROMAXx metal analyser (SPECTRO, Kleve, Germany) using arc/spark optical emission spectrometry (OES).

### 2.2. Low Pressure Die Casting

**Figure 3:**
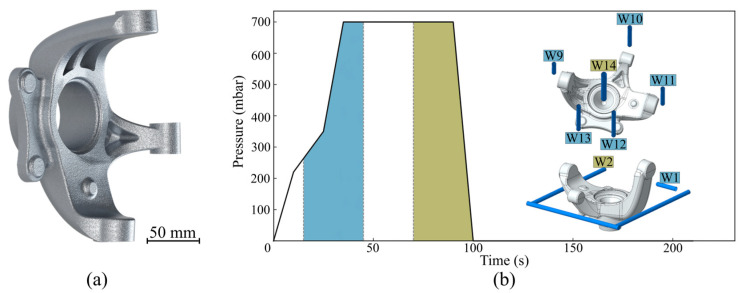
(**a**) Produced steering knuckle’s geometry; and (**b**) LPDC process pressure curves and water-cooling channels usage.

The part to be produced consists of a steering knuckle, with its geometry being represented in Figure 3a. The parts were produced according to the pressure curve presented in Figure 3b, with the concurrent use of the cooling channels (water-cooling, flux around 6 L/s) presented in the figure. The die temperature was measured using a set of thermocouples and averaged around 330–350 °C during the whole casting process, with the die being pre-heated to approximately 350 °C before the commencement of the casting operations. Prior to casting, the die was coated using two layers, a base coating, DYCOTE D R87 (Foseco, Tamworth, UK) and a general-use coating, DYCOTE F34 SA (Foseco, Tamworth, UK). During this process, six parts were produced for posterior characterisation.

### 2.3. Characterisation

**Figure 4:**
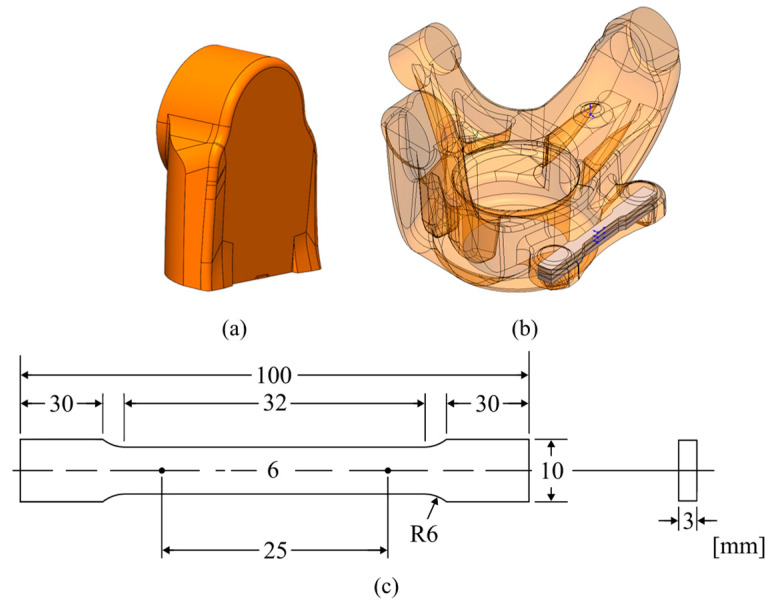
Samples used for characterisation: (**a**) section of the steering knuckle used for hardness testing and optical microscopy observations; (**b**) section of the steering knuckle where the tensile testing specimens were machined from; (**c**) tensile tests specimens’ geometry.

A small section of the casted steering knuckles was cut from one of the arm sections, resulting in the sample illustrated in Figure 4a. In order to evaluate the quality of the castings microstructural, porosity and mechanical properties analysis were performed.

Microstructures were characterised using a Leica DM6 M optical microscope (Leica, Wetzlar, Germany). To expose the different intermetallic compounds, the samples were ground, polished and chemically etched.

An assessment of the porosity presence in the samples was conducted using the LAS X software 5.2.1 (Leica, Wetzlar, Germany), through the lower amplification microstructures obtained using optical microscopy.

Mechanical properties were analysed by macro hardness measurements and tensile testing. The macro hardness measurements were performed utilising a DuraVision 20 G5 semi-automatic hardness tester (ZwickRoell, Ulm, Germany), using the Brinell’s method (tungsten sphere indenter with 2.5 mm of diameter and 31.25 kgf of test load), according to EN ISO 6506-1:2014 standards. Three indentations were performed per sample. The tensile tests were accomplished using an INSTRON 5900R (Instron, Norwood, MA, USA), equipped with an Epsilon Model 4542 axial extensometer (Epsilon Technology, Jackson, WY, USA) with a gage length of 25 mm. A cell load of 100 kN was used for the tests, with a data acquisition rate set to 10 Hz and a constant crosshead speed of 2.5 mm/min. For each casting, three specimens were machined to prepare standard tensile samples (ASTM B557M). The specimens’ dimensions can be visualised in Figure 4c, with the sampling location being presented in Figure 4b.

### 2.4. Numerical Simulation

**Figure 5:**
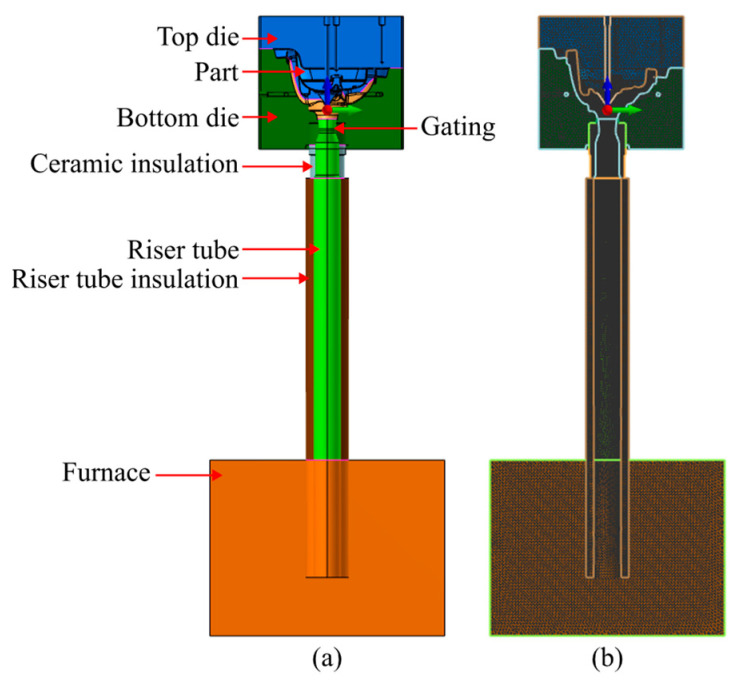
Design of the casting assembly: (**a**) cross-section view in ProCAST highlighting the key volumes of the assembly. (**b**) Cross-section view in ProCAST highlighting the model mesh distribution.

Numerical simulation (flow, solidification, and shrinkage prediction) was performed in order to replicate the experimental trials performed and gain a better understanding of the process, with the experimental conditions used mimicking the experimental conditions experienced. ProCAST^®^ 18.0, (ESI Group, Madrid, Spain) was the selected software to perform finite element analysis. Figure 5a shows the assembly of the elements present in the simulation in ProCAST^®^.

In ProCAST, the assembly follows the mesh size distribution given in Table 2. This element size distribution resulted in a model consisting of 201454 2D elements, 3747796 3D elements and 653276 nodes, as shown in Figure 5b. The chosen mesh density allowed for accurate results with some disregard for computational time as no cyclic simulations were performed.

The alloy used was, as previously mentioned, the AlSi7Mg0.3. In Table 3, the process conditions implemented during the simulation process, including the pouring temperature, initial die temperature, heat transfer coefficients between volumes, and boundary conditions considered are presented.

## 3. Experimental Results and Discussion

### 3.1. Numerical Simulation Results

The simulation began with the smooth filling of the riser tube and gating sections, which continued seamlessly until the 10-s mark, corresponding to the end of the first ramp of the pressure curve. Afterwards, the part filling, corresponding to the second pressure ramp began, allowing for the complete filling of the part section, ensuring minimal turbulence and avoiding the possibility of gas entrapment.

**Figure 6:**
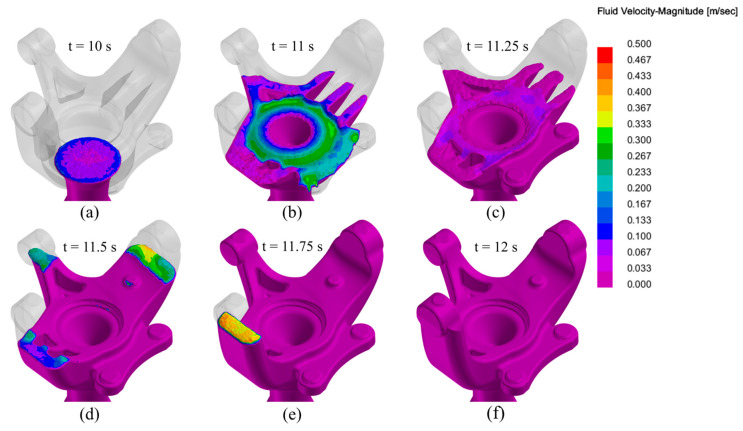
Die filling simulation of the LPDC fabricated AlSi7Mg0.3 aluminium alloy steering knuckle at different times: (**a**) t = 10 s; (**b**) t = 11 s; (**c**) t = 11.25 s; (**d**) t = 11.50 s; (**e**) t = 11.75 s; (**f**) t = 12 s.

Figure 6 showcases various moments of the part filling, allowing for a better comprehension of the flow dynamics inside the part section during the filling process. The process took place smoothly with no turbulence or visible agitation of the melt.

**Figure 7:**
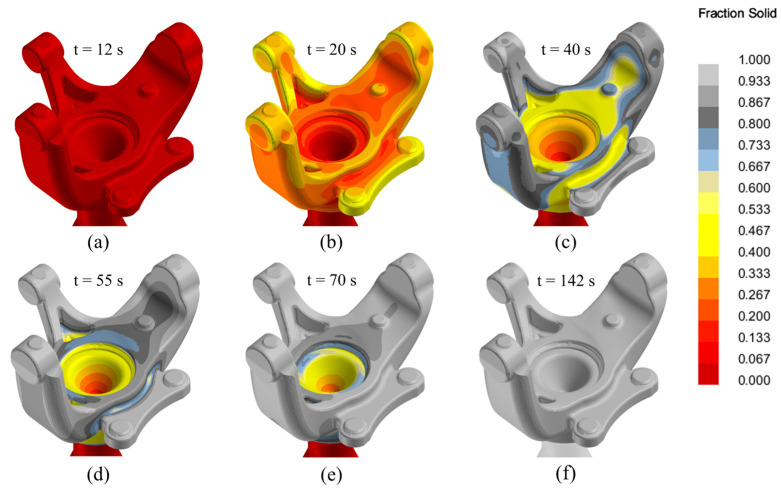
Solidification process simulation of the LPDC fabricated AlSi7Mg0.3 aluminium alloy steering knuckle at different times: (**a**) t = 12 s; (**b**) t = 20 s; (**c**) t = 40 s; (**d**) t = 55 s; (**e**) t = 70 s; (**f**) t = 142 s.

Figure 7 presents the evolution of the solid fraction parameter at various moments during casting solidification, from the completion of filling (12 s) to the complete solidification of the part (142 s). The solidification process began as expected in a top-down progression, with the outer sections of the part solidifying first. At the 100-s mark, the furnace ceased supplying molten metal in accordance with the established pressure curves. The complete solidification of the part section was achieved at the 142 s mark.

**Figure 8:**
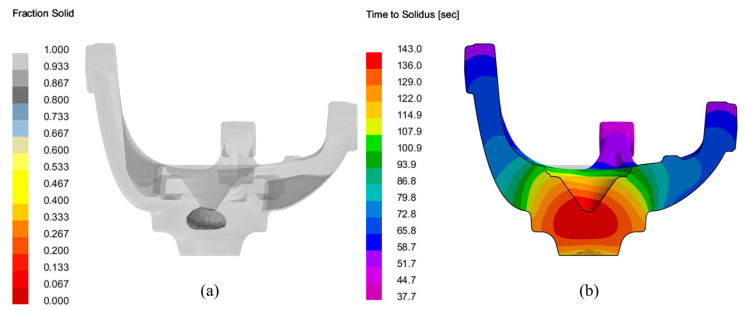
Solidification process simulation of the LPDC fabricated AlSi7Mg0.3 aluminium alloy steering knuckle: (**a**) final section to undergo solidification (cut-off view below 0.9 fraction solid); (**b**) time to solidus cut section view of the steering knuckle.

The simulation shows that the areas adjacent to the gating are, as expected the final areas to undergo solidification, as depicted in Figure 8.

**Figure 9:**
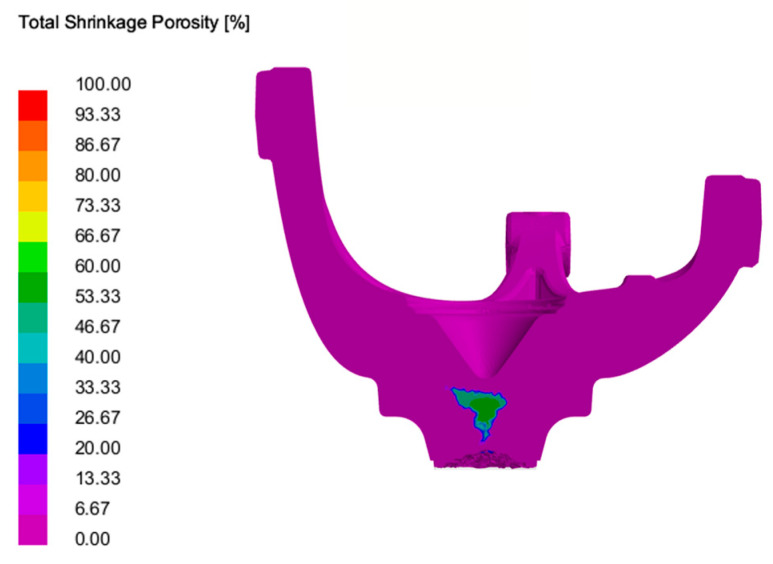
Shrinkage porosity prediction of the LPDC fabricated AlSi7Mg0.3 aluminium alloy steering knuckle.

Regarding the shrinkage porosity analysis, no discernible foci were found during the simulation, as shown in Figure 9, with the areas signalised by the software coinciding with the last areas of the part to undergo solidification, being located in a non-critical section of the part.

### 3.2. Mechanical Properties

#### 3.2.1. Tensile Testing

The attained results for the samples in the six different castings produced were rather similar, with low standard deviation values being achieved when considering all the eighteen tested specimens, per Table 4.

When comparing the obtained results with standardised values (EN 1706), as presented in Table 5 the results fall within the acceptable, showcasing values that tend to the lower end of the spectrum regarding yield stress and ultimate tensile strength, but revelling very satisfactory results regarding total elongation.

**Figure 10:**
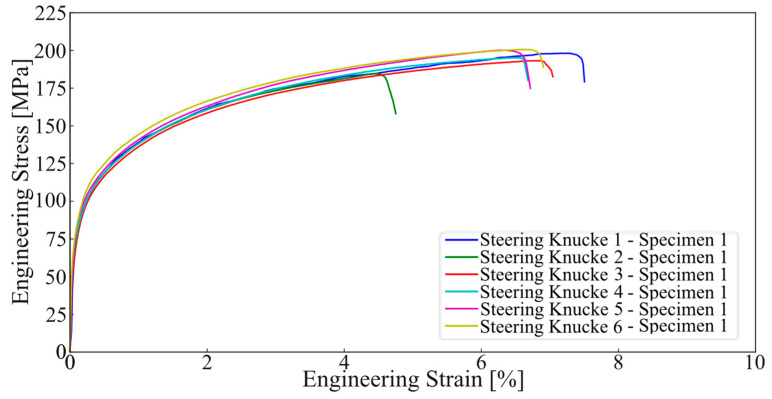
Tensile testing results for all casting specimens 1.

The engineering curve obtained from these tests is presented in Figure 10. The curve represents all the tested samples from the position referent to specimen 1, with consistent behaviour being witnessable across all samples. The curves for the remaining specimens (2 and 3) showed identical behaviours to the one previously addressed.

#### 3.2.2. Hardness Testing

The hardness characterisation of the analysed samples showed a consistent behaviour in similarity to what was observable in the tensile testing, with all the attained results being quite similar, with the average results showing small values of standard deviation. The obtained results are presented in Table 6.

When comparing the obtained results with standardised values (EN 1706) the results are positive, with values over the expected being recorded in all samples. Table 7 showcases this comparison.

### 3.3. Microstructure

**Figure 11:**
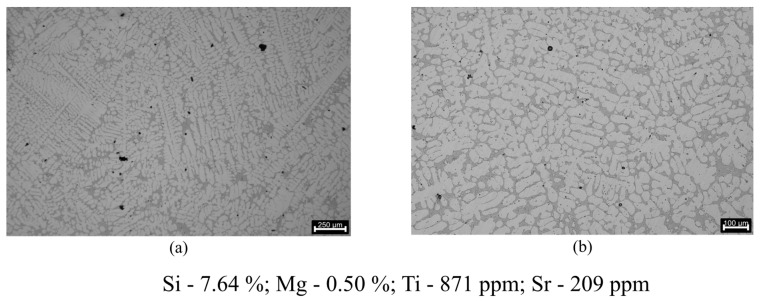
Microstructures regarding the grain refinement evaluation: (**a**) 250 μm scale (22× magnification); (**b**) 100 μm scale (41× magnification).

The microstructure of the AlSi7Mg0.3 alloy in these samples, as shown in Figure 11, comprises a dendritic phase of primary aluminium (α-Al) with both small and large columnar dendritic grains. In some sections, a more equiaxed morphology is also observed.

This irregular structure is expected since no melt refinement procedures like Al5Ti1B master alloy addition for grain refinement were performed [30]. The recorded Ti concentration of 871 ppm further reinforces the fact that no master alloy addition was performed in order to refine the grain, with these values being even below the minimum Ti concentration that is expected on a commercial grade AlSi7Mg0.3 aluminium alloy according to the EN 1676 standard (1000 ppm).

**Figure 12:**
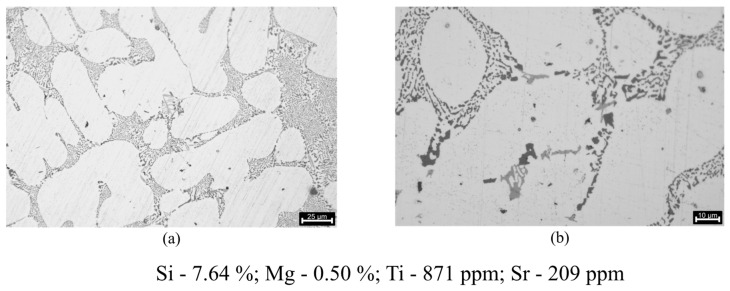
Microstructures regarding the eutectic silicon modification evaluation: (**a**) 25 μm scale (217× magnification); (**b**) 10 μm scale (410× magnification).

Regarding the eutectic Si, in the microstructures analysed in Figure 12, particles that refer to a fruitful eutectic modification are present. The attained eutectic Si structure is between a fully modified structure and a structure absent of lamellae, where the lamellar phase is completely inexistent with only some acicular phase elements being present.

The microstructures further reinforce the successful use of the Al10Sr master alloy, with a very decent level of eutectic modification being achieved. The Sr concentration in the final composition is also very close to the expected with the 250 ppm addition of Al10Sr that was performed, revealing that most likely no interactions between the Sr and other elements in the melt took place.

**Figure 13:**
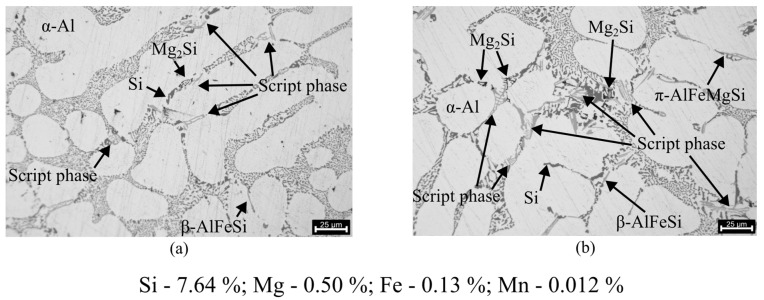
Microstructures regarding the presence of intermetallic phases: (**a**) 25 μm scale (217× magnification); (**b**) 25 μm scale (271× magnification).

The presence of intermetallic phases characteristic of AlSi7Mg0.3 is also visible when analysing the microstructures. Figure 13 illustrates the presence of phases such as α-Al(FeMn)Si and π-AlFeMgSi, with varied Mg_2_Si phases being visible at higher amplifications. The β-AlFeSi phase, which is distinguished by its long, brittle intercepting platelets (more commonly known as needles), which have been demonstrated to exert a negative influence on the properties of Al-Si-Mg alloys [31,32], is less prevalent in the analysed samples. The brittleness and sharp plate-like morphology of this phase can act as stress concentrators, resulting in a significant reduction in the strength, ductility and dynamic fracture toughness of castings [32].

It is important to note that to distinguish between α-AlFeMnSi and π-AlFeMgSi only based on morphology is a very subjective assessment, with the phases in most of the occasions showcasing very similar morphologies. Therefore, in this section, since the specific case where π-AlFeMgSi precipitates from within the β-AlFeSi needles is rarely observable due to the lack of β-AlFeSi, the α-AlFeMnSi and π-AlFeMgSi will be identified as “script phases” [33,34].

After analysing the chemical composition present in the LPDC samples some immediate considerations were made. A high concentration of Mg is present in the composition (0.50%), with this value being even above what is specified in the EN 1706 standard (0.25–0.45%), going in line with the addition that was performed. The slightly higher concentration can be justified with some possible lower-than-expected depletion of Mg in the melt since the melt temperature during treatment was not extremely high.

Regarding the microstructure, as expected, several Mg-rich phases are observable (π-AlFeMgSi and Mg_2_Si), being in accordance with the chemical analysis. A prevalence of the α-AlFeMnSi phase over the β-AlFeSi phase was observable, with the β-AlFeSi phase, with its very distinctive needle-like morphology, being rarely observable. Mn influences the formation of α-AlFeMnSi over β-AlFeSi, which has low adhesion to the matrix and easily allows the formation of the more desirable α-AlFeMnSi phase in the presence of higher amounts of Mn. The presence of the α-AlFeMnSi phase is known to significantly improve the mechanical and corrosion resistance properties of the material [31]. This finding is consistent with the positive mechanical properties observed during mechanical characterisation.

### 3.4. Porosity Analysis

**Figure 14:**
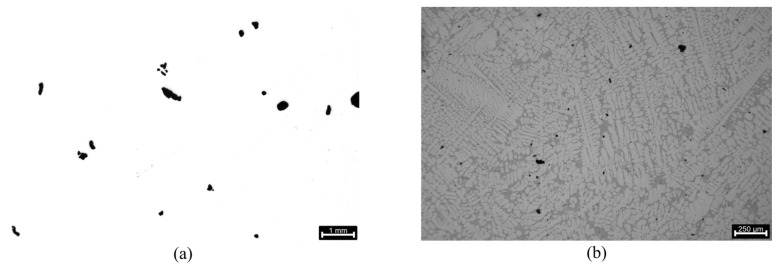
Porosities present in the samples: (**a**) 1 mm scale (5× magnification); (**b**) 250 μm scale (22× magnification).

Some of the porosities found have an irregular shape, which could be consistent with shrinkage porosities resulting from the lack of interdendritic feeding during the solidification process. However, most of the porosities resemble a round/globular shape consistent with gas porosities resulting from the reduction in hydrogen solubility during the aluminium solidification process [35,36]. It is important to note that the porosity focuses are very dispersed and minimal in the area section. Figure 14 displays examples of the porosity morphology at different magnifications, with the results of the porosity analysis being presented in Table 8.

When assessing the total average porosity, it can be concluded that the degassing process was effective, with a porosity area percentage of approximately 0.62% being recorded. With the acceptable range for porosity in gravity die castings using AlSi7Mg0.3 is 0.8–1.1%, these results further reiterate the capability of the LPDC to produce castings with exceptional quality.

An interesting remark can be made regarding the average pore size and roundness assessment. The achieved results show very high standard deviation values, with this occurrence being likely due to the size of the porosities being evaluated, with the margin of error of the software for boundary detection being higher for smaller entities.

The reduced total average roundness could further indicate the presence of shrinkage porosities in the microstructures analysed. However, since the morphology of shrinkage porosities is highly complex and typically non-spherical, unlike gas porosities, it is difficult to assess this topic without performing an X-ray computed tomography (X-CT) analysis to fully visualise the pore geometry [37]. Sphericality is a more reliable indicator of porosity origin than roundness. This is because the roundness value is dependent on the section cut used for microstructural analysis, which can lead to incorrect conclusions.

The results demonstrate the effectiveness of the degassing method employed during the LPDC experimental case. The rotor was found to be a very efficient degassing method, providing excellent degassing results while preserving the aluminium melt’s cleanliness. The foci of possible porosities were notably small.

## 4. Conclusions

In this work, the effects of the LPDC process on the microstructure and mechanical properties together with the analysis of the filling dynamics, solidification process, and presence of shrinkage porosities inherent from the process through the ProCAST 18.0 simulation software of AlSi7Mg0.3 steering knuckles were studied.

1. The use of numerical simulation proved to be valuable as the experimental results confirmed the findings of the simulations. The porosities identified in the experimental work were most likely of gaseous origin (hydrogen porosities), and therefore undetectable by the used software (ProCAST 18.0);
2. The use of a rotor for degassing paired with the LPDC process showed positive results, resulting in total average porosities of 0.62% with very small average pore sizes (0.072 mm^2^);
3. While optical microscopy is an important tool for the microstructural characterisation of castings, it is short-lived and it is necessary to complement it with technologies such as EDS (energy-dispersive X-ray spectroscopy) or SEM (scanning electron microscopy) in order to draw more concrete conclusions, with the results attained on this end in this work being achieved through comparison rather than by analysis;
4. When analysing the results from the LPDC tensile tests and hardness tests, it can be concluded that the produced LPDC castings’ mechanical properties are of acceptable quality, with further work regarding their thermal treatment (T6) being required to evaluate the parts in their full working condition.

## Figures and Tables

**Table 1:** Chemical composition of the AlSi7Mg0.3 alloy (wt.%).

Element	Si	Fe	Cu	Mn	Mg	Zn	Ti	Sr
Standard composition (%) ^1^	6.5–7.5	0.19	0.05	0.10	0.25–0.45	0.07	0.25	-
Actual composition (%)	7.64	0.13	0.03	0.12	0.50	0.01	0.09	0.02

^1^ EN 1706:2020–Aluminium and aluminium alloys–Castings–Chemical composition and mechanical properties.

**Table 2:** Element size distribution.

Tetrahedral Elements Size (mm)	
Riser tube insultation	8
Riser tube	4
Gating	2
Part	2
Bottom die	8
Top die	8
Ceramic insulation	4
Furnace	8

**Table 3:** Simulation process conditions.

Volumes	Materials
Furnace	AlSi7Mg0.3
Part	AlSi7Mg0.3
Gating	AlSi7Mg0.3
Riser tube	AlSi7Mg0.3
Die	Steel H13
Ceramic insulation	Monalite
Riser tube insulation	Refractory fused silica
Pouring temperature (°C)	
720	
**Initial die temperature (°C)**	
350	
Heat transfer coefficient (W/m^2^K^−1^)	Relationship
20	Riser tube–riser tube insulation
20	Furnace–riser tube Insulation
300	Die–ceramic
300	Part–ceramic
300	Gating–ceramic
300	Riser tube–ceramic
500	Ceramic–riser tube insulation
AlSi7Mg0.3–H11 ^1^	Die–part
Boundary Condition	Description
Inlet pressure	Definition of the pressure curves
Mould cooling	Definition of the die cooling mechanism
Exterior heat relationship	Definition of the temperature relationships between the simulation elements and the casting environment (free surface inside the die cavity and the contact of the die with the exterior)

^1^ Heat transfer condition present in the ProCAST^®^ library.

**Table 4:** Mechanical properties obtained for the tested specimens.

Specimen	Yield Stress, Rp0.2 (MPa)	Ultimate Tensile Strength, Rm (MPa)	Total Elongation, At (%)
1_1	94.83	197.91	9.01
1_2	–	Invalid–extensometer slipped	–
1_3	107.88	189.89	6.14
2_1	93.19	184.08	5.71
2_2	102.88	190.16	7.42
2_3	–	Invalid–extensometer slipped	–
3_1	93.48	193.20	8.46
3_2	95.73	184.26	6.48
3_3	98.49	193.46	8.33
4_1	94.13	194.83	8.02
4_2	91.99	194.91	7.98
4_3	99.66	196.20	8.16
5_1	93.33	199.71	8.06
5_2	99.79	198.91	8.66
5_3	96.14	201.58	9.06
6_1	102.33	200.33	8.29
6_2	99.73	200.12	9.36
6_3	103.99	198.85	7.99

**Table 5:** Comparison of the mechanical properties obtained for the tested specimens with the EN 1706 standard reference values.

	Yield Stress, Rp0.2 (MPa)	Ultimate Tensile Strength, Rm (MPa)	Total Elongation, At (%)
LPDC-Average	98.35 ± 4.32	194.90 ± 5.30	7.95 ± 1.01
EN 1706 ^1^	90–150	180–240	4–8

^1^ EN 1706:2020–Aluminium and aluminium alloys–Castings–Chemical composition and mechanical properties.

**Table 6:** Hardness measurement results (values represented in HBW).

Sample	Hardness (HBW)			
	Zone A	Zone B	Zone C	Average
1	73.3	77.1	72.6	74.3 ± 2.0
2	77.3	75.7	73.4	75.5 ± 1.6
3	74.6	78.0	77.8	76.8 ± 1.6
4	76.9	69.4	71.1	72.5 ± 3.2
5	77.5	77.6	78.4	77.8 ± 0.4
6	77.6	76.2	67.2	73.7 ± 4.6
Total Average Hardness:				75.1 ± 3.2

**Table 7:** Comparison of the hardness measurement results obtained for the tested specimens with the EN 1706 standard reference values.

	Hardness (HBW)
LPDC-Average	75.1 ± 3.2
EN 1706 ^1^	50–65

^1^ EN 1706:2020–Aluminium and aluminium alloys–Castings–Chemical composition and mechanical properties.

**Table 8:** Porosity analysis of the optical microscopy results.

Sample	Porosity (%)	Average Pore Size (mm^2^)	Average Roundness
1_1.25x_1	0.96	0.102 ± 0.086	0.49 ± 0.17
2_1.25x_1	0.30	0.043 ± 0.014	0.60 ± 0.23
3_1.25x_1	1.09	0.136 ± 0.075	0.61 ± 0.07
4_1.25x_1	0.25	0.043 ± 0.012	0.59 ± 0.19
4_1.25x_2	0.94	0.073 ± 0.041	0.65 ± 0.11
4_1.25x_3	0.40	0.050 ± 0.019	0.55 ± 0.15
4_1.25x_4	1.03	0.066 ± 0.039	0.71 ± 0.14
4_1.25x_5	0.74	0.045 ± 0.012	0.65 ± 0.20
5_1.25x_1	0.95	0.076 ± 0.061	0.56 ± 0.23
6_1.25x_1	0.59	0.072 ± 0.027	0.64 ± 0.16
6_1.25x_2	0.47	0.066 ± 0.019	0.49 ± 0.09
6_1.25x_3	0.73	0.058 ± 0.026	0.61 ± 0.14
6_1.25x_4	0.12	0.057 ± 0.015	0.47 ± 0.08
6_1.25x_5	0.12	0.060 ± 0.016	0.48 ± 0.10

## Data Availability

Data are contained within the article.
